# St. Vitus Dance

**Published:** 1886-04

**Authors:** 


### ST. VITUS'S DANCE.

St. Vitus's Dance or *Chorea,* is characterized by involuntary convulsive motions of the voluntary muscles, causing remarkable grimaces and contortions, without pain or fever. Sometimes it is confined to particular parts, and is generally more on one side of the body than the other. This affection was very prevalent near Ulm in the fifteenth century, and on the first of May every year the sufferers made a pilgrimage to the chapel of St. Vitus, to pray for restoration to health. As the worst cases produce violent motions, these pilgrims were said to dance in the chapel like insane people, hence the name St. Vitus's Dance. It very rarely attacks any but young persons between the ages of eight and thirteen. Its causes have never been discovered, the hearty and robust being as liable to it as the pale and sickly; but it has sometimes been ascribed to fright. It generally commences slowly, with slight twitching of the face and fingers, extending by degrees to the lower extremities. The motion of the voluntary muscles ceases to be under command, the limbs are agitated with violent, singular movements, various muscles are in constant motion, contortions of the countenance are frequent, the patient walks as if palsied, and cannot do anything with the arms without moving them in an irregular, zigzag manner. If a cup of drink be put into the hand, it cannot be carried to the mouth, without convulsive difficulty, accomplishing it at last as if by accident, and drinking with greedy haste. It always continues for some weeks, often gets nearly well in a month or two, but somes lasts a long time. Good wholesome living, bathing, suitable clothing, or a long sea voyage and change of climate, are the most likely to mitigate and cure it.

**How to Obtain Old Age.**—How to live long and healthy is embraced by M. Revielle, of Paris, in four general rules of living:

1. How to live.
2. Know thyself.
3. Arrange wisely the habits of life.
4. Combat disease in its beginning.

Canarvo, an Italian of noble birth, noted alike for his learning and age, in early youth wandered from his father's house and prostituted himself till life was despaired of by himself and his physician. At the age of 25 he came to himself, and by a properly regulated life lived to be 100 years old. He gives this one universal rule: "Properly protected from excessive cold and heat, live soberly and temperately."

